# 1242. Advanced Automated Analysis System for Antimicrobial Stewardship Programs Including Clinician-focused Features Developed with Artificial Intelligence tools in a Resource-Limited Setting.

**DOI:** 10.1093/ofid/ofad500.1082

**Published:** 2023-11-27

**Authors:** Ivan Felipe Gutierrez-Tobar, J U A N BRAVO, M A R T H A HERNANDEZ, N I D I A TURMEQUE, Juan Pablo Londono-Ruiz, C A R L O S ALVAREZ

**Affiliations:** Clínica Infantil Santa Maria del Lago y Clinica Infantil Colsubsidio, Bogota, Distrito Capital de Bogota, Colombia; CLINICA INFANTIL SANTA MARIA DEL LAGO, Bogota, Distrito Capital de Bogota, Colombia; CLINICA INFANTIL SANTA MARIA DEL LAGO, Bogota, Distrito Capital de Bogota, Colombia; CLINICA INFANTIL SANTA MARIA DEL LAGO, Bogota, Distrito Capital de Bogota, Colombia; Clinica Infantil Colsubsidio, Staphylored Colombia, Bogota, Distrito Capital de Bogota, Colombia; Departamento Enfermedades Infecciosas, Clínica Colsanitas, Universidad Nacional de Colombia, BOGOTA, Distrito Capital de Bogota, Colombia

## Abstract

**Background:**

Antimicrobial Stewardship Programs (ASP) are a priority for the control of bacterial resistance. Artificial intelligence (AI) provides tools that can enable the development and optimization of strategies to optimize ASPs. This abstract describes the experience of improving ASP using AI tools, including a component that facilitates the analysis of antibiotic prescriptions to the ASP and a component to provide real-time feedback and training to clinicians.

**Methods:**

AI tools were used to develop code that integrated a centralized prescription database with local recommendations for automatic analysis. Combination of AI tools, Excel, Google Sites, Apps Script, Google Forms, and Data Studio, were used to optimize ASP technologies to develop real-time ASP analysis visualization and feedback dashboards for prescribers. (Graph 1)

**Figure:**
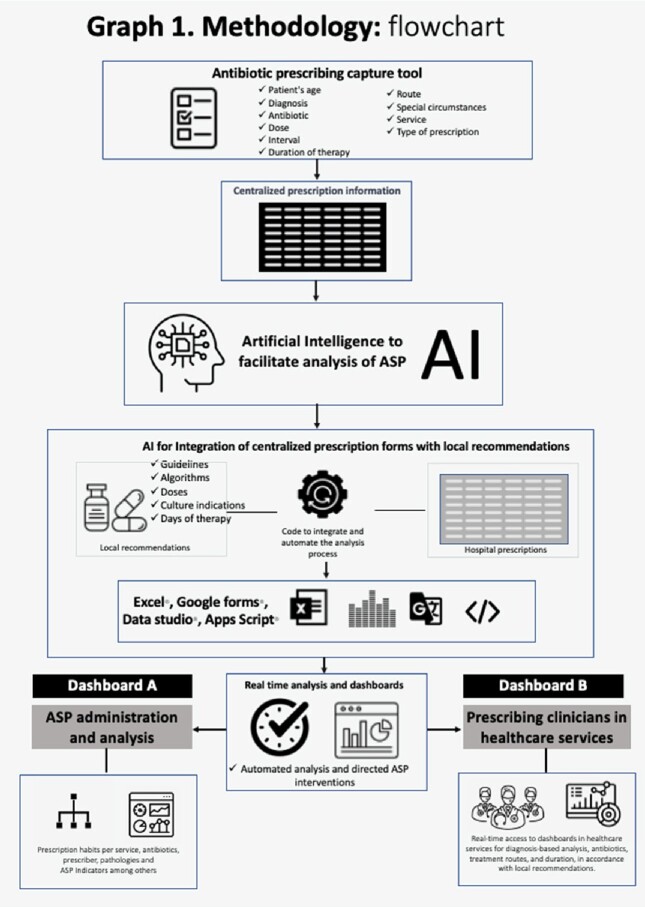

**Results:**

A control panel was created in Google Sites to access various ASP visualization sections. Different dashboards were developed for ASP analysis, including daily prescription analysis, analysis by service, pathology, prescriber profile, and by the most prevalent infections. Interfaces and forms were created to register ASP analysis and interventions.

Broad-spectrum and daily prescribed antibiotics dashboards allow the identification of prescription characteristics and the results of the automated evaluation (adherence to guidelines and dosages). A specific dashboard allows for real-time analysis of the main ASP metrics. Additionally, dashboards were created for clinicians to provide real-time automated recommendations for switching to oral administration, duration according to guidelines, and other factors based on the registered diagnosis, antibiotics, dosage, and days. (Graph 2-3)

**Graph 2.**
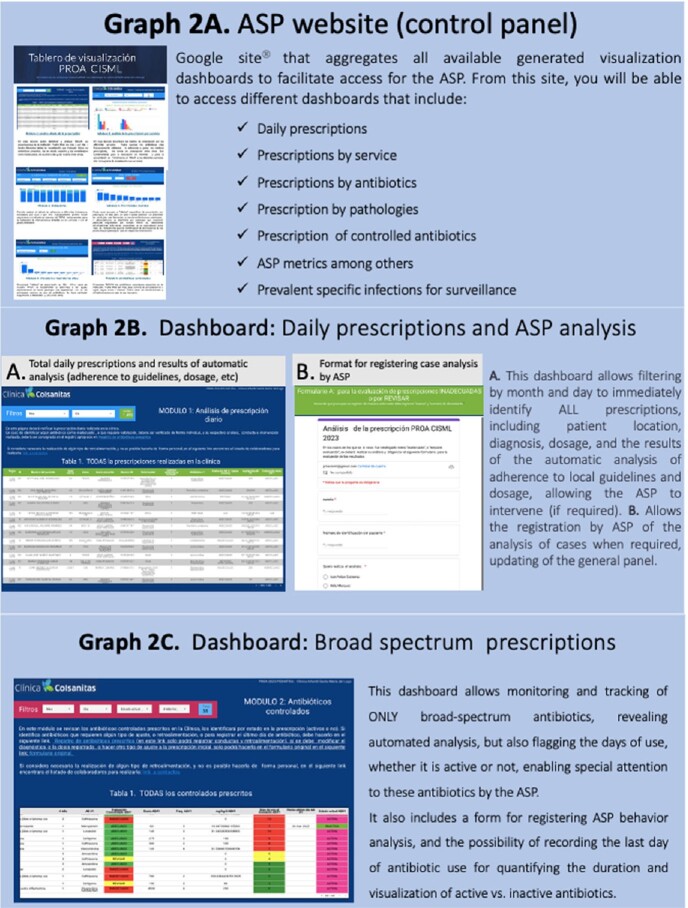
ASP website (control panel)

Google site that aggregates all available generated visualization dashboards to facilitate access for the Antimicrobial Stewardship Program.

**Graph 3.**
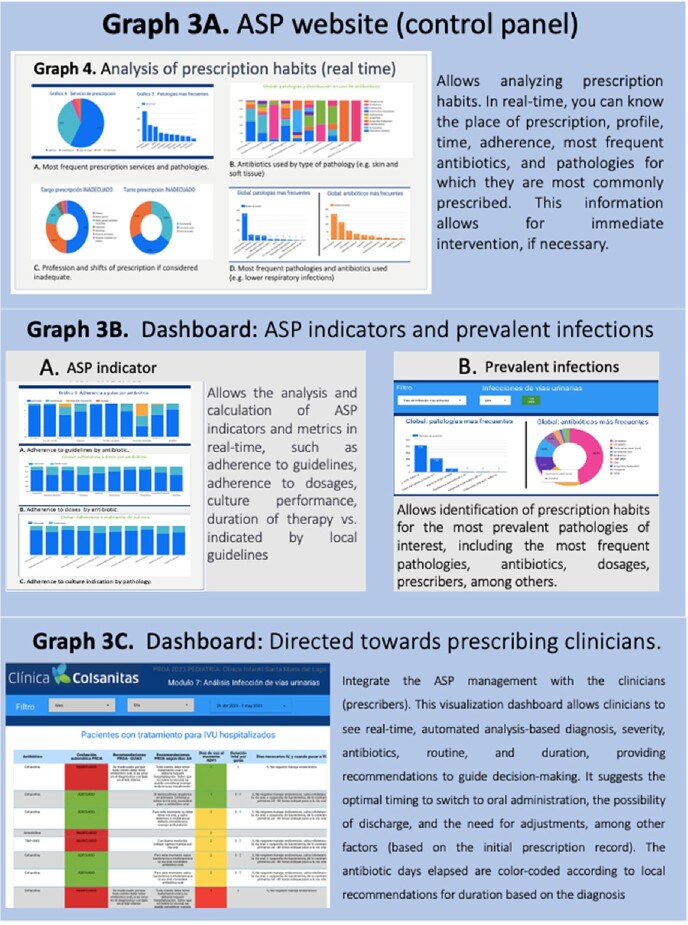
Dashboard: Daily prescriptions

This dashboard allows filtering by month and day to immediately identify ALL prescriptions, including patient location, diagnosis, dosage, and the results of the automatic analysis of adherence to the guide and dosage, allowing the ASP to intervene immediately if required.

Conclusions

**Figure:**
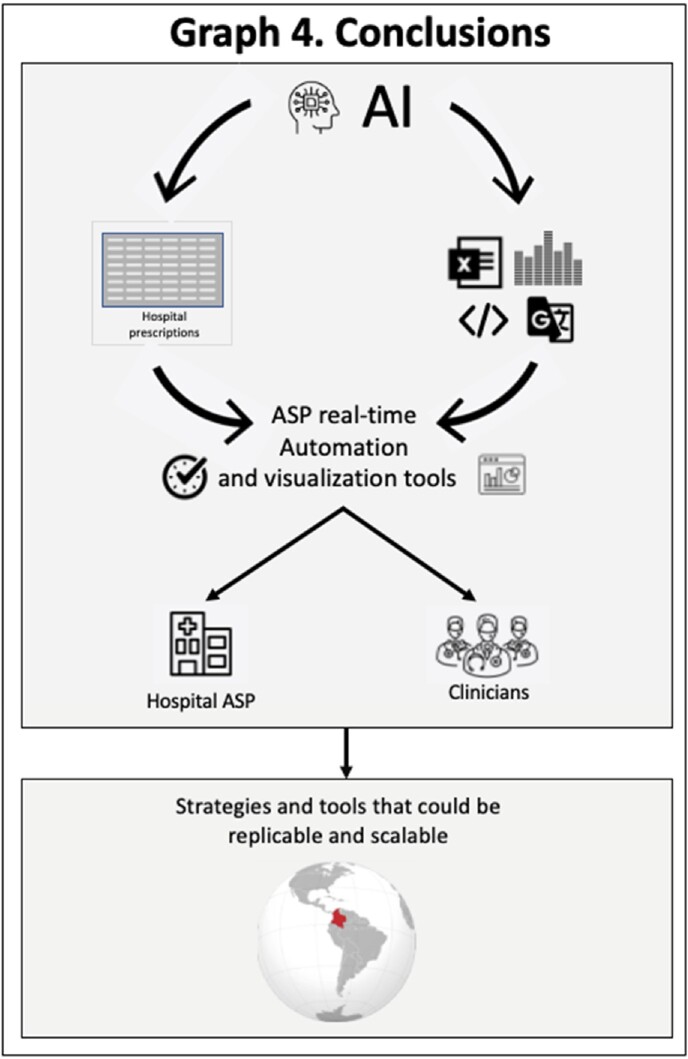

A tool for systematizing an ASP using AI strategies can optimize ASPs in institutions with limited resources and can be adapted for use in other scenarios.

**Conclusion:**

Use of AI tools in ASP automation has led to the development of a systematic tool for analyzing antibiotic prescriptions. This innovative approach provides a practical solution for developing and optimizing ASP in resource-limited environments. Additionally, it incorporates a new element that includes visualization dashboards for clinicians to receive real-time feedback and suggestions about antimicrobial prescriptions. This tool could be replicable and scalable for possible use in other scenarios. (Graph 4)

**Disclosures:**

**All Authors**: No reported disclosures

